# Essential genome of *Campylobacter jejuni*

**DOI:** 10.1186/s12864-017-4032-8

**Published:** 2017-08-14

**Authors:** Rabindra K. Mandal, Tieshan Jiang, Young Min Kwon

**Affiliations:** 1 Department of Poultry Science, University of Arkansas, Fayetteville, AR 72701 USA; 2 Cell and Molecular Biology Program, University of Arkansas, Fayetteville, AR 72701 USA; 3 Present Address: Department of Microbiology and Immunology, Clinical Translational Research Building, University of Louisville, Louisville, KY 40202 USA

**Keywords:** *Campylobacter*, Transposon sequencing (Tn-seq), Essential genes

## Abstract

**Background:**

*Campylobacter* species are a leading cause of bacterial foodborne illness worldwide. Despite the global efforts to curb them, *Campylobacter* infections have increased continuously in both developed and developing countries. The development of effective strategies to control the infection by this pathogen is warranted. The essential genes of bacteria are the most prominent targets for this purpose. In this study, we used transposon sequencing (Tn-seq) of a genome-saturating library of Tn5 insertion mutants to define the essential genome of *C. jejuni* at a high resolution*.*

**Result:**

We constructed a Tn5 mutant library of unprecedented complexity in *C. jejuni* NCTC 11168 with 95,929 unique insertions throughout the genome and used the genomic DNA of the library for the reconstruction of Tn5 libraries in the same (*C. jejuni* NCTC 11168) and different strain background (*C. jejuni* 81–176) through natural transformation. We identified 166 essential protein-coding genes and 20 essential transfer RNAs (tRNA) in *C. jejuni* NCTC 11168 which were intolerant to Tn5 insertions during in vitro growth. The reconstructed *C. jejuni* 81–176 library had 384 protein coding genes with no Tn5 insertions. Essential genes in both strain backgrounds were highly enriched in the cluster of orthologous group (COG) categories of ‘Translation, ribosomal structure and biogenesis (J)’, ‘Energy production and conversion (C)’, and ‘Coenzyme transport and metabolism (H)’.

**Conclusion:**

Comparative analysis among this and previous studies identified 50 core essential genes of *C. jejuni*, which can be further investigated for the development of novel strategies to control the spread of this notorious foodborne bacterial pathogen.

**Electronic supplementary material:**

The online version of this article (doi:10.1186/s12864-017-4032-8) contains supplementary material, which is available to authorized users.

## Background

*Campylobacter* species are a leading cause of bacterial foodborne illness worldwide and one of the most common infectious agents of the last century. Despite a reduction in the incidence of infections by a number of major foodborne pathogens due to global efforts, *Campylobacter* infections have continued to increase in both developed and developing regions across the globe including the U.S., Europe, Australia, Africa, Asia and the Middle East. Additionally, *Campylobacter* harbors antimicrobial resistance genes with the capability of horizontal transfer between pathogenic and commensal microorganisms, which could possibly lead to the emergence of multidrug resistant microorganisms. Hence, researchers speculate that *Campylobacter* will continue to remain a threat to global public health for years to come [1, 2]. The current issue warrants a multifaceted approach to intervene and control *Campylobacter* infections, including identification of indispensable essential genes that are related to basic cellular functions or metabolic pathways/processes. These genes have the potential of becoming novel targets for the development of new antibiotics or vaccines.

Essential genes are defined as those that are absolutely required for the viability of cellular life [3, 4]. Experimental techniques, such as single-gene knockouts [5–7], transposon mutagenesis [8, 9], and antisense RNA and RNA interference [10, 11] have been used to identify essential genes. In addition, computational approaches to track down essential genes involve comparative genomics, supervised machine learning, constraint-based methods, and integrative genomics approaches based on orthology and phylogeny [12–16]. However, the most reliable method used to define the essential genome is transposon mutagenesis via transposon sequencing (Tn-seq). The basic principle of this approach involves the creation of a transposon insertion library in the bacteria of interest and identification of individual transposon-genome junction sites on a global scale by the Tn-seq method. This process helps identify virtually all dispensable genes, which allows for the identification of the entire set of essential genes in the genome simultaneously in a single experiment by negative selection of transposon mutants [17]. Methods with minor variations are known as InSeq [18], TraDIS [19], HITS [20], Tn-seq circle [21], Tn-seq [22], and RB-TnSeq [23]. Recently, Hutchison III et al. (2016) has used improved transposon mutagenesis methods for the identification of quasi-essential genes, which were then used as a basis to create a minimal synthetic bacterial genome, *Mycoplasma mycoides* JCVI-syn3.0, smaller than the genomes of any autonomously replicating cell found in nature [24].

Stahl and Stintzi (2011) identified 195 essential genes of *Campylobacter jejuni* (*C. jejuni*) NCTC 11168 required for growth at 37 °C under a microaerophilic atmosphere on a rich Muller-Hinton medium with 7201 individual mutants (Tn5) using a microarray-based approach for tracking transposon insertions [25]. Furthermore, Metris et al. (2011) also identified 233 essential genes of *C. jejuni* strain NCTC 11168 strain based on a total of 9550 transposon insertions in the genome using two different transposons (Mariner and Tn7) on Blood Agar Base no.2 (Oxoid) plates supplemented with 5% *v*/v defibrinated horse blood at 42 °C under microaerophilic conditions [26]. More recently, Gao et al. (2014) identified 175 essential genes of *C. jejuni* 81–176 based on ~50,000 transposon insertion mutants of *C. jejuni* 87–176 on brucella agar plates at 37 °C in 10% CO_2_ atmosphere [27]. However, these studies have had only a limited overlap in their lists of essential genes, probably because of the different culture conditions for recovery of the mutants, different strain backgrounds, varying levels of saturation of transposon insertions, or different analytical approaches. Since these approaches for essential gene discovery are based on the identification of the genomic regions that do not tolerate transposon insertions, the accuracy of essential gene discovery should be critically dependent on the saturation level of the transposon insertion library.

In this study, we created a highly complex Tn5 mutant library of *C. jejuni* NCTC 11168 (seed library) with more than 95,000 unique insertions in the genome. *C. jejuni* NCTC 11168 required 166 essential protein coding genes for the growth on Muller-Hinton (MH) agar at 37 °C under microaerophilic conditions. Additionally, we reconstructed a Tn5 mutant library in the same (*C. jejuni* NCTC 11168) and a different strain background (*C. jejuni* 81–176) by transferring the insertions in the seed library to the recipient cells via natural transformation to develop and validate a powerful approach for comparative functional genomics of *C. jejuni*.

The reconstruction technique of a Tn5 library in *Campylobacter* developed in this study has several advantages. First, the viability of *Campylobacter* strains normally reduces significantly over time even during storage at −80 °C for both the wild type and mutant strains. Additionally, this reduction in viability can be more severe depending on the mutations the mutant strains carry. This presents the challenge of storing the mutant library without altering the representativeness of the viable mutant strains. However, if we could reconstruct the mutant library in a wild type background without significant changes in the representativeness of the mutations present, it would be an important advancement in functional genomics. Second, the ability to reproduce the mutant library by natural transformation with genomic DNA of a complex library would facilitate the use of the DNA as an important resource for functional genomic analysis of *C. jejuni* among different labs. Third, the reconstruction of the library will facilitate comparative genomic analysis across different strains, including different deletion mutant backgrounds. For example, this reconstruction method can be employed to perform genetic interaction mapping by introducing the same library into wild type and different deletion mutants of the same strain background, followed by Tn-seq analysis.

Finally, we combined all existing data from the previous and current studies to define a core set of essential genes of *C. jejuni.*

## Results and discussion

### Evaluation and comparison of the libraries based on Illumina sequencing data

We generated complex library of *C. jejuni* NCTC 11168 following natural transformation of in vitro mutagenized genomic DNA with commercially available Tn5 transposome complex kit (EZ-Tn5™ < KAN-2 > Tnp Transposome™ Kit). Fourteen natural transformations were performed, each producing ~100,000 mutants and combined to create a complex library that contains a total of 1.4 million mutants. Previously, various strategies have been attempted for efficient transposon mutagenesis of *C. jejuni*. For in vivo mutagenesis, the preformed Tn5 transposome complex was introduced into the various *C. jejuni* strains via electroporation [28]. This strategy yielded ~3000 random mutants per electroporation for *C. jejuni* strain 81–176, but the efficiency was extremely low for other strains tested, limiting the application of the approach. On the contrary, for in vitro mutagenesis, transposition reactions were conducted using genomic DNA of *C. jejuni*, purified transposon sequence plus purified transposase enzyme of either Tn5 [29] *mariner* [30]or Tn552 [31]. Then the in vitro mutagenized DNA was used to transform *C. jejuni* cells through natural transformation, yielding 3000–7000 transposon mutants per reaction. In the current study, we achieved an efficiency of transposon mutagenesis far higher than previously reported (100,000 vs. 3000–7000 transposon mutants per transformation). We speculate that the high efficiency in our study was due to the use of a preformed transposome complex for in vitro mutagenesis of genomic DNA as compared to adding transposase and transposon sequences separately into the reaction, as has been done in all previous studies for in vitro transposon mutagenesis in *Campylobacter* [29–31].

**Fig. 1:**
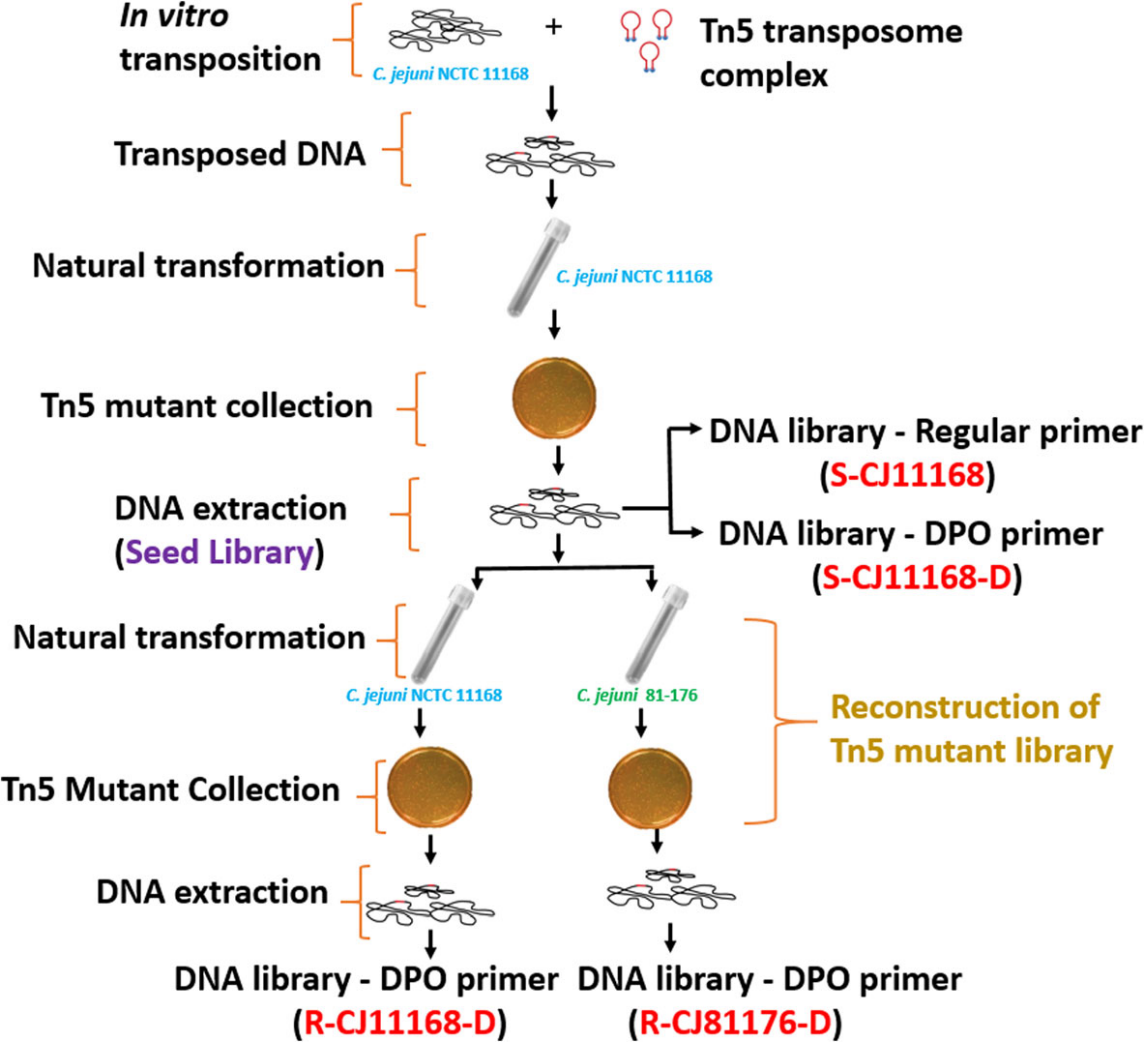
Design of experiment. EZ-Tn5™ < KAN-2 > Tnp Transposome™ Kit was used for in vitro transposition of genomic DNA of *C. jejuni* NCTC 11168. The transposed DNA was then naturally transformed into *C. jejuni* NCTC 11168 and mutants were cultured on MH agar with TMP and Km (Seed library: S-CJ11168). Seed library DNA was extracted and used for the reconstruction of the Tn5 library in the same (*C. jejuni* NCTC 11168: R-CJ11168-D) and different strain backgrounds (*C. jejuni* 81–176: R-CJ81176-D). Regular and DPO primers were used for linear extension to make DNA libraries for Illumina sequencing. [S: the seed library, R: the reconstructed library, D: dual priming oligonucleotide (DPO), CJ11168: *C. jejuni* NCTC 11168; and CJ81176: *C. jejuni* 81–176]

Equal volumes of the mutant pools were combined to make a seed library (S-CJ11168). Genomic DNA from the seed library was used for reconstruction of the Tn5 mutant library. We collected 281,000 mutants from the natural transformation of the seed library in the same strain background (*C. jejuni* NCTC 11168: R-CJ11168-D) and 82,000 mutants in a different strain background (*C. jejuni* 81–176: R-CJ81176-D) as shown in Fig. 1 and Table 1.

**Table 1:** Overview of *C. jejuni* Tn5 mutant libraries

Library	# Tn5 Mutants	Total Reads	Mapped Reads (%)	# Unique Insertions	Mean (± SE)
S-CJ11168	1,400,000	9,040,241	6,812,731 (75.36)	95,929	71.02 ± 0.90
S-CJ11168-D	1,400,000	6,920,934	6,448,244 (93.1)	79,178	81.44 ± 11.54
R-CJ11168-D	281,000	6,052,446	5,685,107 (93.93)	52,607	108.07 ± 13.89
R-CJ81176-D (a)	82,000	1,638,463	1,303,248 (79.54)	29,565	44.08 ± 5.89
*a- > CJ11168			1,493,694 (91.16)	32,623	45.79 ± 5.46

Mean is the average reads per unique insertion in the Tn5 library with standard error (SE)

S: Seed library, R: Reconstruction, D: DPO, CJ11168: C. jejuni NCTC 11168, CJ81116: C. jejuni 81–176

*Tn5 library (R-CJ81176-D, a) when mapped against the donor strain *C. jejuni* NCTC 11168 genome (a- > CJ11168), a higher alignment rate was achieved (91.6%)

Transposon junction sequences were amplified using two different primers i.e. regular and dual priming oligonucleotide (DPO) in the linear extension step with downstream processes remaining the same for all DNA libraries. The regular primer was used for linear extension of the seed library in *C. jejuni* NCTC 11168 (S-CJ11168) while the DPO primer was used for the seed library in *C. jejuni* NCTC 11168 (S-CJ11168-D), the reconstructed library in same strain background *C. jejuni* NCTC 11168 (R-CJ11168-D), and the other strain background *C. jejuni* 81–176 (R-CJ81176-D). The DPO primer is believed to block mismatched priming, thereby accomplishing higher PCR specificity [32]. The site of Tn5 insertion in each Tn5 library was determined through next-generation sequencing on the HiSeq platform. Demultiplexing Illumina sequencing reads without any mismatch in the barcode sequence and Tn5 mosaic end produced 9,040,241 reads for S-CJ11168; 6,920,934 reads for S-CJ11168-D; 6,052,446 reads for R-CJ11168-D and 1,638,463 reads for R-CJ81176-D. Similarly, R-CJ1168-D had the highest number of reads per unique insertion site and R-CJ81176-D had the lowest reads per unique insertion site with S-CJ11168 having the highest and R-CJ81176-D having the lowest median reads per unique insertion site (Table 1).

Next, the 22 bp transposon genome junction sequence reads were mapped to their respective genome using the default parameter of Bowtie2.2.8, which reports the best alignment. It is interesting to note that *C. jejuni* NCTC 11168 has 30.6% GC content [33], however, the GC content of the 22 bp genomic sequence across all the Tn5 libraries was 40.25% (SE ± 2.22). This might reflect the preference of Tn5 transposition into guanosine (G) and cytidine (C) rich sequences [34]. The overall alignment rate was 85.50% (SE ± 5.46). The regular primer had a significantly lower alignment rate when compared to the DPO primer library (75.36% vs. 92.87% respectively), clearly indicating a higher specificity in binding the target DNA by the DPO primer. It was also observed that the regular primer produced a lower standard error (SE) with mean reads per unique insertion when compared to the DPO primer (0.90 vs. 11.54, respectively, for S-CJ11168 library). Thus, from this data, we can conclude that the DPO primer produced the Tn-seq amplicon libraries are more representative of the Tn5 mutant pools than those produced using the regular primer based on the higher alignment rate (Table 1).

Furthermore, the seed libraries S-CJ11168 and S-CJ11168-D had the most unique insertions throughout the genome, with 95,920 and 79,178 unique insertions respectively; followed by the reconstructed library in same strain background (R-CJ11168-D: 52,607) and the fewest in the other strain background (R-CJ81176-D: 29,565) (Table 1). *C. jejuni* NCTC 11168 libraries had 47,090 shared unique insertions genome-wide. Importantly, only 2218 (2.1%) of the Tn5 insertion sites were unique to the reconstructed library of *C. jejuni* NCTC 11168 (R-CJ11168-D). Similarly, the seed library amplified using the regular and the DPO primer had 73,649 (71%) unique insertions in common (Additional file 1: Figure S1). The fact that a significantly lower number of unique insertions was detected in R-CJ11168-D in comparison to S-CJ11168-D (52,607 vs. 79,178) may be due to the insufficient number of Tn5 mutants (281,000 mutant colonies) collected during the reconstruction experiment. The considerably lower number of unique insertions in R-CJ81176-D in comparison to R-CJ11168-D (29,565 vs. 52,607) is likely due to the genomic differences in the two strains but may also be due to the insufficient number (82,000 mutants) of Tn5 mutants collected to form the reconstructed library R-CJ81176-D. The saturation level of reconstructed libraries could possibly be increased by increasing the number of Tn5 mutants by carrying out more natural transformations of the seed library.

### Identification of essential gene in *Campylobacter jejuni* NCTC 11168

**Fig. 2:**
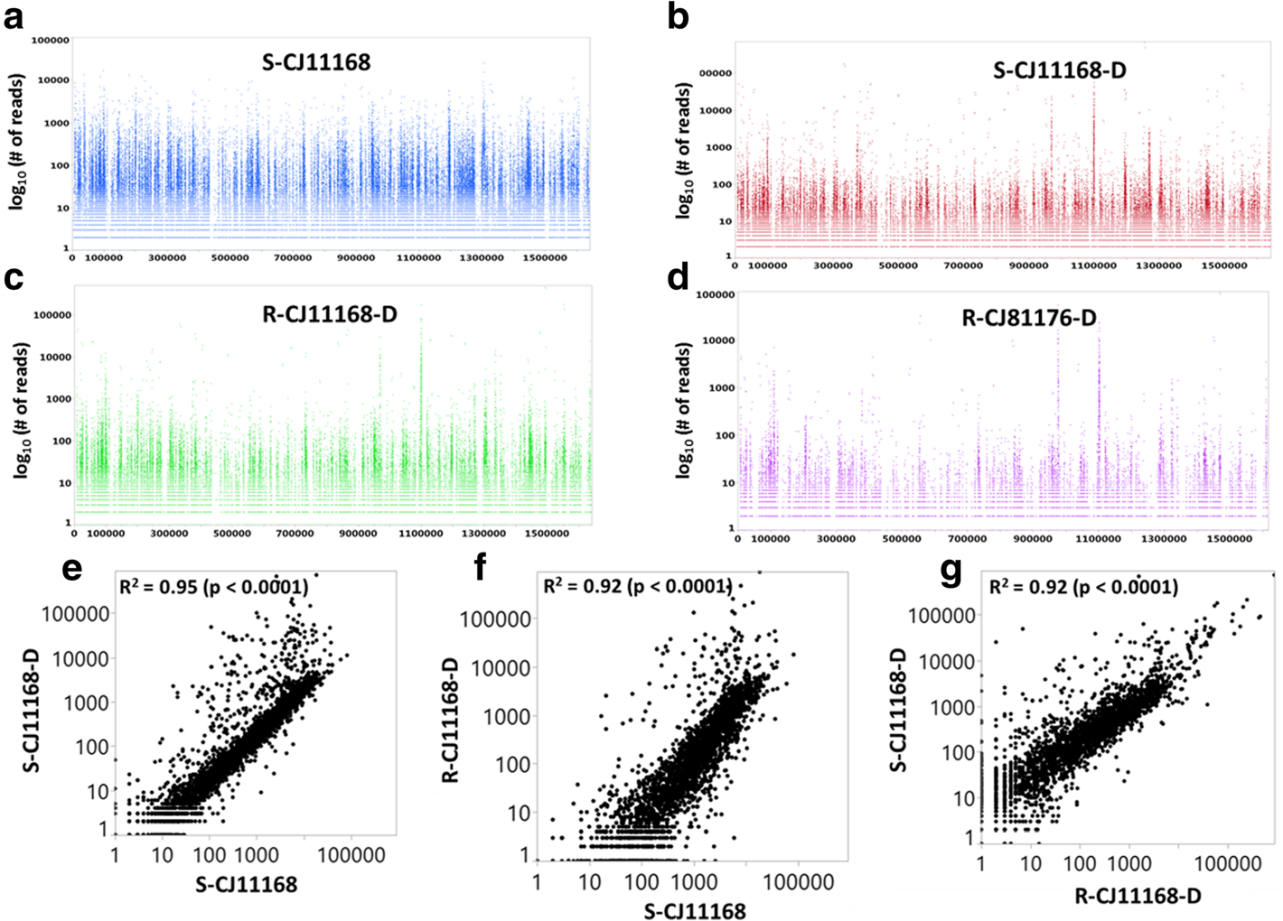
Overview of Illumina sequencing of *C. jejuni* Tn5 libraries. Read distribution of Tn5 mutant libraries of *C. jejuni*: (**a**) S-CJ1118, (**b**) S-CJ11168-D, (**c**) R-CJ11168-D and (**d**) R-CJ81176 (X-axis: Genomic coordinate of *Campylobacter*; Y-axis: log_10_ transformed read counts). Spearman correlation (R^2^) of Tn5 reads distribution based on a 400 bp window size between different libraries of *C. jejuni* NCTC 11168: (**e**) S-CJ11168 vs S-CJ11168-D, (**f**) S-CJ11168 vs R-CJ11168-D and (**g**) R-Cj11168-D vs S-CJ11168-D. Tn5 libraries are named as previously

The EL-ARTIST pipeline was used for the identification of essential genes of *C*. *jejuni* required for optimal growth on MH agar plates under microaerophilic conditions at 37 °C. The *Campylobacter* Tn5 libraries had no noticeable replication bias in the reads distribution throughout the genome (Fig. 2a, b, c, and d). Replication bias is usually suggested by a ‘V’ shaped reads distribution with a higher number of reads at the origin of replication. Absence of a replication bias in *C. jejuni* NCTC 11168 might be due to the higher doubling time (112 min) when grown in MH broth as opposed to lower doubling times of some bacteria that have a replication bias, such as *V. cholera* which has a doubling time of 16–20 min when grown in rich media [35, 36]. Read counts of Tn5 libraries were mapped to 400 bp genomic windows against the *C. jejuni* NCTC 11168 genome. A high Spearman correlation was observed between the seed library prepared with the regular (S-CJ11168) and the DPO primer (S-CJ11168-D) based on read counts binned using a 400 bp window size (R^2^ = 0.95, *p* < 0.0001) as shown in Fig. 2e. A slightly lower Spearman correlation (R^2^ = 0.92, *p* < 0.0001) was observed between the reconstructed library in the same genetic background (R-CJ11168-D) with the seed libraries prepared with the regular (S-CJ11168) and the DPO primer (S-CJ11168-D) as shown in Fig. 2f and g, respectively.

Tn5 sequencing analysis using the EL-ARTIST pipeline revealed ~250 genes essential in *C. jejuni* NCTC 11168 required for optimal growth on rich MH agar under microaerophilic conditions at 37 °C. The seed library *C. jejuni* NCTC 11168 (S-CJ11168) identified 280 essential genes (15 domain essential and 265 entirely essential genes) for optimal growth. Likewise, the same seed library prepared with DPO primer (S-CJ11168-D) identified 278 essential genes (9 domain essential and 269 entirely essential genes) and the reconstructed library in the same genetic background (R-CJ11168-D) revealed 284 essential genes (18 domain essential and 266 entirely essential genes) using the same conditions. All three of these libraries shared 200 common essential genes (Additional file 1: Figure S2).

However, when the Tn5 insertions were examined closely at the gene level, multiple genes identified as essential by EL-ARTIST had significant sequence reads corresponding to the insertions in the genes. For example, *spoT* had 64 Tn5 unique insertions with 358 reads (in S-CJ11168 library) that was called as essential for optimal growth by EL-ARTIST. Furthermore, the EL-ARTIST pipeline was not sensitive enough to pick up the genes with less than a 400 bp window size. In addition, windows far smaller than 400 bp, for instance 100 bp, can give false positive results due to a lack of insertions in many 100 bp windows.

**Fig. 3:**
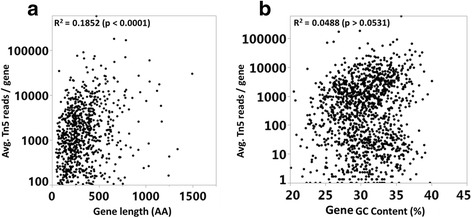
Tn5 insertion bias analysis in Tn5 libraries of *C. jejuni* NCTC 11168. Spearman correlation (R^2^) between (**a**) gene length (amino acid length) and average Tn5 insertion reads and (**b**) GC content (%) of all genes and average Tn5 insertion reads. Average Tn5 reads is the average of Tn5 reads in the central 80% of gene in all the 3 libraries of *C. jejuni* NCTC 11168 (S-CJ11168, S-CJ11168-D, and R-CJ11168-D)

Bioinformatics pipeline analysis results are subject to variation depending upon the algorithm and statistical power. To overcome the noticed limitation of EL-ARTIST in analyzing our dataset, the same dataset was re-analyzed to identify the essential genes according to the definition of essential genes as those that cannot tolerate any insertions. The genes meeting this condition for the internal 80% of the coding region in all three Tn5 libraries of *C. jejuni* NCTC 11168 were considered to be essential for growth or survival in this study. We identified 166 essential coding sequences (CJ-11168) of *C. jejuni* NCTC 11168 with no Tn5 insertions (Additional file 2: SH2). 52.4% of the essential genes of *C. jejuni* NCTC 11168 were on the negative strand while 47.5% were on the positive strand. Genes that contain even one insertion can be labeled as non-essential, but genes lacking insertions cannot be necessarily classified as essential due to sequence bias of Tn5 insertions and the smaller genes having a lower chance of transposon insertion [26]. However, in this study, there was a significantly low correlation between the Tn5 insertion read counts and gene length (Spearman correlation = 0.1852, *p* < 0.0001) (Fig. 3a). Also, Tn5 transposons are inserted randomly throughout the entire genome with some preference towards GC rich DNA sequences [34]. Nevertheless, we did not observe any correlation between the Tn5 read insertion in the central 80% of genes (CDS) and GC content (%) of the entire gene (CDS) as shown in Fig. 3b (Spearman correlation = 0.0488, *p* > 0.0531).

Furthermore, to back up our analysis at the gene level, we looked for the Tn5 insertion in pseudogenes of *C. jejuni* NCTC 11168. Out of a total of 38 pseudogenes in the entire genome of *C. jejuni* NCTC 11168, all of the pseudogenes had Tn5 insertions in at least one of the seed libraries of *C. jejuni* NCTC 11168 (Additional file 2: SH4). This also indicates the high coverage of Tn5 mutagenesis in the seed library. Nonetheless, the seed library S-CJ11168 (sequenced with the regular primer) and S-CJ11168-D (sequenced with the DPO primer) missed Tn5 insertions in only one pseudogene each, Cj0740 and Cj0742, respectively. However, the reconstructed library in the same background (R-CJ11168-D) had no Tn5 insertions in 11 pseudogenes. This is most likely due to the low saturation level in the reconstructed libraries, which was likely caused by the limited number of transposon mutants collected to form the reconstructed libraries. This explanation is supported by the significantly low level of unique insertions in the reconstructed libraries in comparison to the seed libraries (Table 1). This shortcoming may be able to be mitigated by increasing the number of Tn5 mutants recovered to form reconstructed libraries and ensuring a similar level of sequencing depth across the libraries that are compared.

**Fig. 4:**
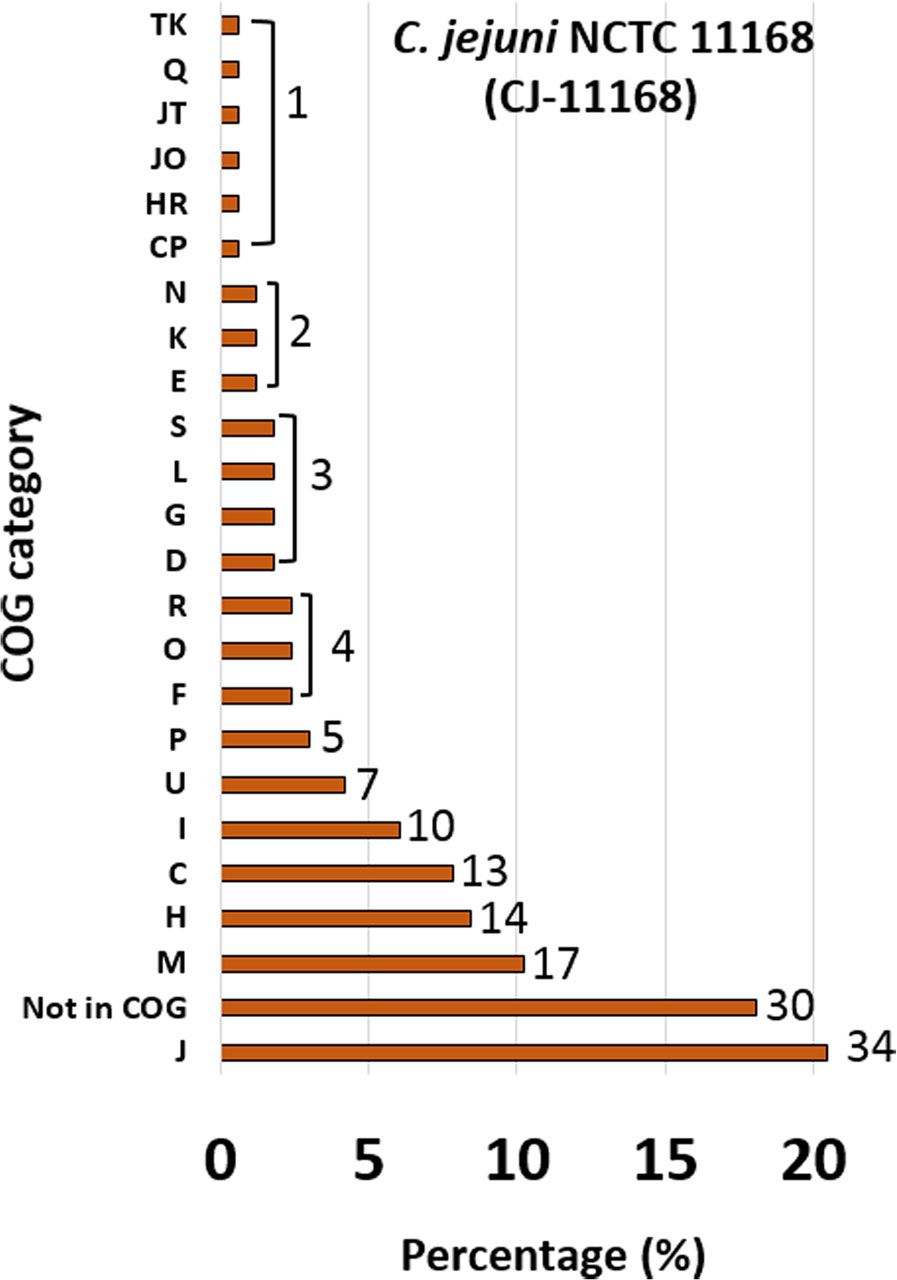
Cluster of orthologous group **(**COG) categories of essential genes of *C. jejuni* NCTC 11168 (CJ-11168)**.** Essential genes were defined as genes without any Tn5 insertions in central 80% of genes among all the three libraries of *C. jejuni* NCTC 11168 (S-CJ11168, S-CJ11168-D, and R-CJ11168-D). The number at the top of bar is the number of essential genes in that COG category. J- Translation, ribosomal structure and biogenesis; X– Not in COG; M- Cell wall/membrane/envelope biogenesis; H- Coenzyme transport and metabolism; C- Energy production and conversion; I- Lipid transport and metabolism; U- Intracellular trafficking, secretion, and vesicular transport; P– Inorganic ion transport and metabolism; F- Nucleotide transport and metabolism; O- Post-translational modification, protein turnover, and chaperones; R- General function prediction only; D– Cell cycle control, cell division, chromosome partitioning; G- Carbohydrate metabolism and transport; L- Replication, recombination and repair; S- Function unknown; E- Amino acid transport and metabolism; K- Transcription; N- Cell motility; V- Defense mechanisms; Q- Secondary metabolites biosynthesis, transport, and catabolism; and T- Signal transduction mechanisms

Next, we assigned essential genes to Cluster of Orthologous Groups (COG) using the NCBI FTP site (ftp://ftp.ncbi.nlm.nih.gov/genomes/archive/old_refseq/Bacteria/). The COG categories highly enriched among the 166 essential genes were: J- Translation, ribosomal structure and biogenesis (20.48%), Not in COG (18.07%), M- Cell wall/membrane/envelope biogenesis (10.24%), H- Coenzyme transport and metabolism (8.43%), C- Energy production and conversion (7.83%) and I- Lipid transport and metabolism (6.02%). The COG moderately enriched were: U- Intracellular trafficking, secretion, and vesicular transport (4.22%), P– Inorganic ion transport and metabolism (3.01%), F- Nucleotide transport and metabolism (2.41%), O- Post-translational modification, protein turnover, and chaperones (2.41%), and R- General function prediction only (2.41%). Also, the low abundant COG categories with only one gene each were CP, HR, JO, JT, Q (Secondary metabolites biosynthesis, transport, and catabolism) and TK (Fig. 4). Similar to our findings, the most commonly enriched COG in essential genes of other bacteria like *Porphyromonas gingivalis*, *Herbaspirillum seropedicae*, *Vibrio cholera*, *Rhodopseudomonas palustris*, *Burkholderia cenocepacia*, and the synthetic bacteria *Mycoplasma mycoides* JCVI-syn3.0 were related to translation, ribosomal structure and biogenesis (J), cell wall/membrane/envelope biogenesis (M), and coenzyme transport and metabolism (H) [8, 9, 24, 37–39].

Interestingly, we identified 20 essential transfer RNA (tRNA) out of a total of 43 tRNA genes in the entire genome of *C. jejuni* NCTC 11168 and none of the ribosomal RNA genes encoding 16S ribosomal RNA, 23S ribosomal RNA, and 5S ribosomal RNA were identified as essential (Additional file 2: SH3). The 20 essential tRNA genes are those that are required for the transfer of arginine, asparagine, aspartic acid, glutamic acid, glycine, leucine, lysine, methionine, selenocysteine, serine, tryptophan, tyrosine, and valine during protein synthesis. Another essential ncRNA was *rnpB* which is a component RNA of the ribonuclease P enzyme (RNase P) together with the RnpA protein (also identified as an essential gene in our study) functions in the processing of 4.5S RNA and tRNA precursor molecules in *E. coli* (https://ecocyc.org/gene?orgid=ECOLI&id=EG30069). Similar to our findings, in a recent study, Rosconi et al. (2016) found 22 tRNA genes and one 23S ribosomal RNA gene to be essential in *Herbaspirillum seropedicae*, an endophyte that colonizes crops like rice and maize, during in vitro growth in TY medium [38].

### Reconstruction of insertion libraries in a different strain background (*C. jejuni* 81–176)

We transformed the seed library genomic DNA through natural transformation into a different strain background (*C. jejuni* 81–176). Homologous recombination of the seed library genomic DNA in a different strain requires significant homology between the incoming DNA fragments and the target regions at the DNA level. *C. jejuni* strains have a high level of genomic diversity, and Poly et al. (2005) previously reported that *C. jejuni* 81–176 had 63 kb of new chromosomal DNA sequences unique to this strain, which contains 86 open reading frames, when compared to *C. jejuni* NCTC 11168, based on a shotgun DNA microarray analysis [40]. To identify the genomic region in the donor strain with significant homology to the recipient genome, DNA sequences that consist of 1 kb flanking upstream and downstream of each coding sequence (CDS) in *C. jejuni* NCTC 11168 along with the CDS were BLASTed against *C. jejuni* 81–176. This analysis produced 1535 best BLAST hits based on the highest bit score for each query sequence. Further filtering (described in the Materials and Methods section) resulted in 904 query genes with substantial similarity of which 895 are orthologous genes commonly present in both *C. jejuni* 11,168 and *C. jejuni* 81–176 genomes (Additional file 3: SH1). This process was designed to eliminate the genes that are unique to the *C. jejuni* 81–176 genome from the downstream analysis so that we can prevent or minimize the false identification of the genes with zero Tn5 insertions as essential genes, where the absence of insertion is in fact due to the lack of identity.

Out of 895 orthologous genes with significant similarity including 1 kbp flanking sequences, 384 genes had zero Tn5 insertions (Additional file 3: SH2) and are thus considered to be essential genes required for the in vitro growth of *C. jejuni* 81–176 on rich MH agar at 37 °C under microaerophilic conditions. These 384 essential genes (CJ-81176) of *C. jejuni* 81–176 were categorized into cluster of orthologous groups (COGs). The highly enriched COGs were: J- Translation, ribosomal structure and biogenesis (16.67%), Not in COG (13.28%), C- Energy production and conversion (7.55%), H- Coenzyme transport and metabolism (6.25%), R- General function prediction only (5%), E- Amino acid transport and metabolism (5.73%), S- Function unknown (4.95%), M- Cell wall/membrane/envelope biogenesis (4.43%), and F- Nucleotide transport and metabolism (4.17%). Other moderately enriched COGs ranging from 3.5% to 2.5% in abundance were: posttranslational modification, protein turnover, chaperones (O), inorganic ion transport and metabolism (P), replication, recombination and repair (L), intracellular trafficking, secretion, and vesicular transport (U), cell cycle control, cell division, chromosome partitioning (D), carbohydrate transport and metabolism (G), lipid transport and metabolism (I); and transcription (K). The other COGs with 1 or 2 genes were 12.50% of the total number of genes (Additional file 1: Figure S3).

**Fig. 5:**
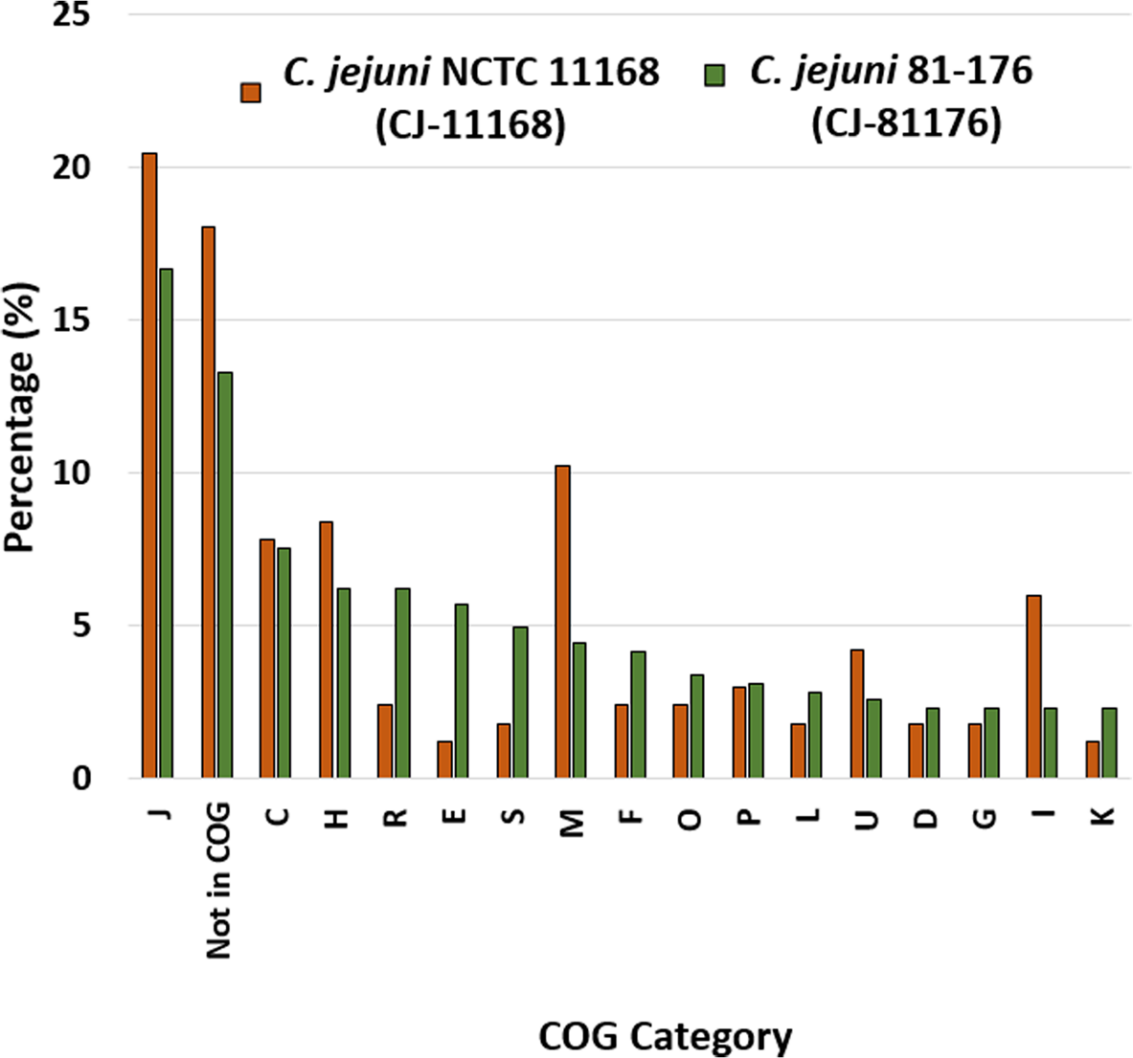
Comparison of major COG categories enriched in the essential genome of *C. jejuni* NCTC 11168 (CJ11168) and *C. jejuni* 81–176 (CJ81176). COG categories annotations are the same as in Fig. 4

Both strains, *C. jejuni* NCTC 11168 and *C. jejuni* 81–176, had similar levels of enrichment in the COGs including: nucleotide transport and metabolism (F), transcription (K), replication, recombination and repair (L), posttranslational modification, protein turnover, chaperones (O), cell cycle control, cell division, chromosome partitioning (D), carbohydrate transport and metabolism (G), inorganic ion transport and metabolism (P), energy production and conversion (C), and intracellular trafficking, secretion, and vesicular transport (U) with differences in relative abundance ranging from −1.76% to +1.62%. The COG category such as ‘Amino acid transport and metabolism’ (E) was relatively higher in *C. jejuni* 81–176 by 4.53% than in *C. jejuni* NCTC 11168, and ‘Lipid transport and metabolism’ (I) and ‘Cell wall/membrane/envelope biogenesis’ (M) were higher in *C. jejuni* NCTC 11168 by 3.68% and 5.81% respectively than in *C. jejuni* NCTC 81–176 (Fig. 5). These differences may have been due to the considerable variation in the genomic backgrounds of the two strains, which in turn likely reflects differences in the regulatory networks and their responses to environmental stimuli such as the availability of nutrients and temperature. In addition, the fact that the essential genes in *C. jejuni* 81–176 were identified only from the genomic regions common in both strains could have resulted in some bias in the overall enrichment procedure.

### Comparative study

**Fig. 6:**
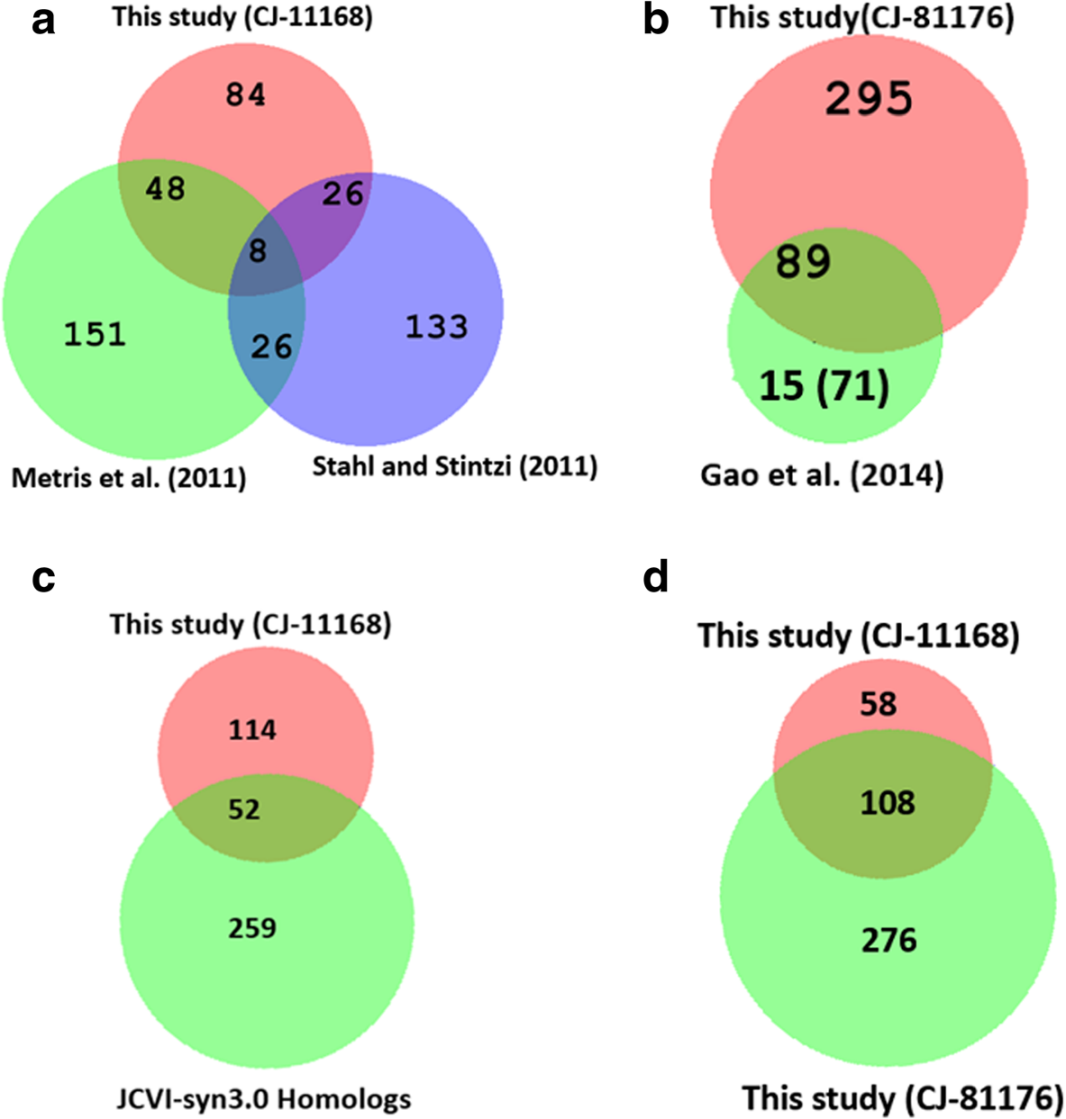
Venn diagrams indicating the numbers of shared essential genes of *C. jejuni* found between this and previous studies. **a** Essential genes of *C. jejuni* NCTC 11168 common between Metris et al. (2011), Stahl and Stintzi (2011) and this study (CJ-11168). **b** Essential genes of *C. jejuni* 81–176 common between Gao et al. (2014) and this study (CJ-81176). Number of genes inside small bracket did not have highly homologous sequences in the *C. jejuni* NCTC 11168 background according to our criteria. **c** Common genes shared between this study (CJ-11168) and homologous proteins of JCVI-syn3.0 against *C. jejuni* NCTC 11168. **d** Common essential genes between the two *Campylobacter* strains identified in this study (CJ-11168 - *C. jejuni* NCTC 11168 and CJ-81176 - *C. jejuni* 81–176)

Two research groups have previously reported on the essential genes in *C. jejuni* NCTC 11168 on a genome-wide scale. Surprisingly, there is only a limited correlation between the essential genes compliment identified in the reports by Metris et al. (2011) and Stahl and Stintzi (2011). We compared the essential genes of *C. jejuni* previously identified by Metris et al. (2011) and Stahl and Stintzi (2011) with the findings in our current study. Since the level of library saturation is very much higher in our study, we expected that our result would show significantly higher overlaps with each of these previous reports. However, there were limited overlaps in the essential genes of *C. jejuni* NCTC 11168 among the two previous studies and our current study as shown in Fig. 6a. Likely explanations are: 1) a limited number of transposon mutants (~ 10,000 mutants) in previous studies, 2) differences in growth conditions: 37 °C vs. 42 °C, and MH agar vs. Blood agar, and 3) techniques used for transposon insertion site mapping: microarray used in previous studies vs. next-generation sequencing in this study. These arguments are also substantiated by Gao et al. (2014).

Interestingly, substantial overlapping was observed between the essential genes of *C. jejuni* 81–176 identified in this study and the previous report by Gao et al. (2014) as shown in Fig. 6b, despite the considerable differences in experimental procedures. Twenty-three percent of essential genes from this study were common to Gao et al. (2014) and more than 50% of the genes identified by Gao et al. (2014) were identified in our study. The primary source of variation with the previous study (Gao et al. 2014) was the method of transposon library construction. In our study, genomic DNA from the *C. jejuni* NCTC 11168 Tn5 library was naturally transformed into *C. jejuni* 81–176 to create the Tn5 mutant library. Thus, Tn5 mutagenesis occurred only at the genomic loci in *C. jejuni* 81–176 with a significant amount of homology between the two strains.

Additionally, we were interested in comparing our results with the essential genes used for the creation of JCVI-syn3.0, the first synthesized minimal bacterium with the smallest genome (473 genes) capable of self-replication in laboratory media. *C. jejuni* NCTC 11168 protein coding genes were searched for homologous proteins against the JCVI-syn3.0 genome using BLASTp with a similarity score cutoff of 1e^−5^ and 311 homologous proteins were found in *C. jejuni* NCTC 11168. Among the 311 homologous proteins of *C. jejuni*, 52 genes encoding these proteins overlapped with the essential genes of *C. jejuni* NCTC 11168 identified in our study (Fig. 6c). As expected, little overlap was seen between the two distantly related bacteria probably due to the significant differences in their genetic networks and physiology.

However, there was substantial overlap between essential genes of *C. jejuni* NCTC 11168 and *C. jejuni* 81–176 (Fig. 6d). Approximately, 65% of the essential genes of *C. jejuni* NCTC 11168 were common to *C. jejuni* 81–176. In contrast, fewer essential genes of *C. jejuni* 81–176 were shared (~28%) with *C. jejuni* NCTC 11168. This could be partly due to the assumption that the genes with no Tn5 insertion are essential genes, which may have led to the inclusion of some nonessential genes in the list of essential genes for *C. jejuni* 81–176. This difference may be also due to a lack of sufficient nucleotide sequence identity between genes in the two different strain backgrounds of *C. jejuni*, despite extensive filtering for homologous sequences to reduce the noise in the data analysis. Another important factor that may have contributed to the identification of false essential genes in *C. jejuni* 81–176, may be the insufficient number of Tn5 mutants collected during the reconstruction of the library.

Notably, the essential genes of *C. jejuni* NCTC 11168 identified in this study had an extensive homolog hits in the Database of Essential Genes (DEG, http://www.essentialgene.org. DNA sequences of 166 essential genes of *C. jejuni* NCTC 11168 were found using BLASTx among the essential genes from 46 bacterial species in the database with default parameters (Expect - 1E-05, Score - 100, and Matrix –BLOSUM62). Out of 166 essential protein-coding genes of *C. jejuni* NCTC 11168, 135 genes had homologs among the DEG and 2879 DEG genes had homology with our essential genes of *C. jejuni* NCTC 11168. Most of the essential genes with no hits in the DEG were hypothetical proteins (15 genes). However, six integral membrane proteins (Cj0369c, Cj0423, Cj0430, Cj0544, Cj0564, and Cj0851c), three periplasmic proteins (Cj0659c, Cj0854c, and Cj0114), and other genes (*pseH* and *rnpA*) were also essential genes in this study with no hit in the DEG.

**Fig. 7:**
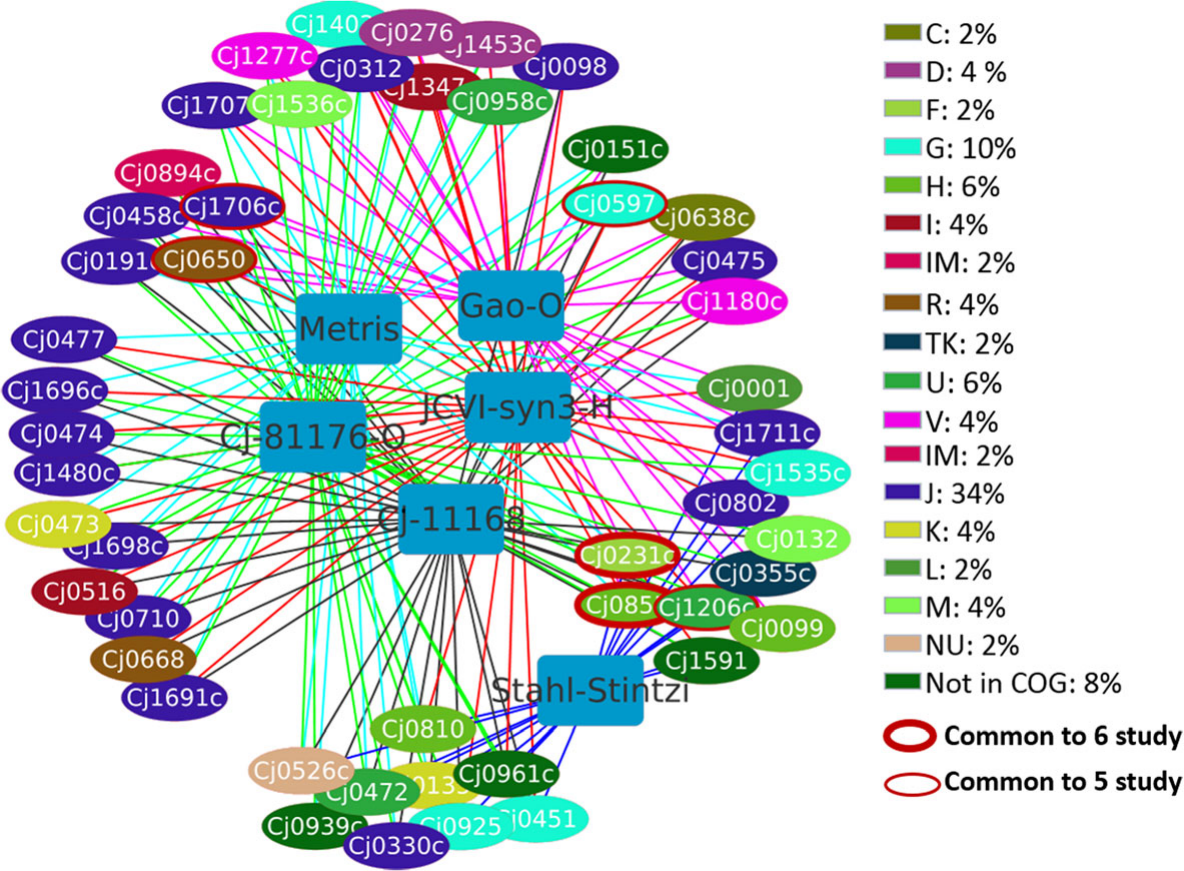
Core essential genes of *C. jejuni* NCTC 11168. Genes are colored to indicate the COG category. Numbers after the legend indicate percentages of the COG category enriched in the core essential gene list. Soft edge rectangle- various studies: Metris [26], Stahl-Stintzi [25], Gao-O [27], JCVI-syn3-H [24], CJ-81176-O: *C. jejuni* 81–176 (this study), and CJ-11168: *C. jejuni* NCTC 11168 (this study). O and H in the study name indicate orthologs and homologs respectively. Genes circled in thick red and thin red are common in six and five studies, respectively. All the genes in this network were identified as essential in at least four of the studies

Next, we studied the core essential genes of *C. jejuni* through comparative analysis of all essential genes identified in this study (*C. jejuni* NCTC 11168 and *C. jejuni* 81–176), previous studies by Metris et al. (2011), Stahl and Stintzi (2011), Gao et al. (2014), and those in the synthetic bacterium, JCVI-syn3.0 (Fig. 7 and Additional file 4). Essential genes of C. *jejuni* 81–176 orthologous to *C. jejuni* NCTC 11168 strain and proteins of JCVI-syn3.0 homologous to *C. jejuni* NCTC 11168 were considered. There were 50 genes common to the six studies with each of these genes being shared amongst at least four of the studies (Fig. 7). Most of these genes belonged to COG category ‘translation, ribosomal structure and biogenesis’ (J; 34%), ‘carbohydrate metabolism and transport’ (G; 10%), followed by genes ‘Not in COG’ (8%), ‘coenzyme transport and metabolism’ (H; 6%) and ‘intracellular trafficking, secretion, and vesicular transport’ (U; 6%). The importance of category J being a large portion of the core essential genes is further supported by the fact that ribosomal proteins are the most prominent drug targets in bacteria that have been used to control infections [41].

NrdF and *folD* were identified as essential in all of the above 6 studies while *ftsY*, *fba*, *engB* and *rplD* were identified as essential in 5 of the studies (Fig. 7 and see Additional file 4 for corresponding gene name and locus_tag). A live attenuated *Salmonella* Typhimurium *aroA* vector expressing the *Mycoplasma hyopneumoniae* ribonucleotide reductase R2 subunit (NrdF) gene was shown to induce a cell-mediated immune response [42]. A deletion mutant of *ftsY*, the gene encoding a signal recognition particle protein, in *Streptococcus pneumonia* induced potent serotype-independent protection against otitis media, sinusitis, pneumonia and invasive pneumococcal disease [43]. *fba* encodes the class II fructose 1,6-bisphosphate aldolase enzyme, which is important for bacterial, fungal and protozoan glycolysis and gluconeogenesis and is considered as a putative drug target against *Mycobacterium tuberculosis*, the causative agent of tuberculosis [44].

Many other essential *Campylobacter* genes shared among at least 4 of the above studies have been used as drug targets and vaccine constructions to mitigate several bacterial infections. BirA, a biotin protein ligase, is an emerging drug target against *E. coli* and other pathogens such as *Staphylococcus aureus* and *M. tuberculosis*. Inhibition of *lpxC*, an enzyme that has a role in lipid A biosynthesis, by a small-molecule antibiotic in *Acinetobacter baumannii*, protected mice from infection by modulation of inflammation and enhancing opsonophagocytic killing [45]. MreB, a rod shape-determining protein, when blocked by MreB-specific antibiotics inhibited the growth of *Chlamydia* [46]. IspH (4-hydroxy-3-methylbut-2-enyl diphosphate reductase) satisfied all criteria of being a putative drug target against *Corynebacterium pseudotuberculosis*, a pathogenic bacterium that causes caseous lymphadenitis (CLA), ulcerative lymphangitis, mastitis, and edematous in a broad spectrum of hosts [47]. KsgA (rRNA small subunit methyltransferase A) has been associated with clarithromycin resistance in *M. tuberculosis* [48]. A GalU (UTP-glucose-1-phosphate uridylyltransferase) mutation in *Francisella tularensis*, the causative agent of tularemia, was protective against homologous challenge in mice [49]. GapA (glyceraldehyde 3-phosphate dehydrogenase) was also used in the construction of an effective DNA vaccine against *Haemophilus parasuis*, the causative agent of swine polyserositis, polyarthritis, and meningitis [50].

## Conclusion

We constructed an unprecedentedly complex Tn5 library of *C. jejuni* NCTC 11168 with more than 95,000 unique insertions in the genome. The genomic DNA of the seed library was effectively used for reconstruction of a Tn5 library in the same strain background (*C. jejuni* NCTC 11168) and with limited success in a different strain background (*C. jejuni* 81–176). Despite discrepancies among studies, comparative analysis conducted in this study showed the essential genes of *C. jejuni* were also found to be essential in other bacterial species and many of the genes have been exploited as drug target and in vaccine development against a wide range of bacterial diseases. By definition, the absence of essential genes have the potential to severely affect the survival of a bacterium, therefore, these genes can be further exploited to develop novel strategies to curb this important foodborne pathogen.

## Methods

### Bacteria strains and growth conditions

*C. jejuni* NCTC11168 and *C. jejuni* 81–176 were grown on Muller-Hinton (MH) agar plates at 37 °C under microaerophilic conditions (O_2_–5%, CO_2_–10%, and N_2_- Balance). Trimethoprim (TMP, 10 μg/ml,) and Kanamycin (Km, 50 μg/ml) were added to the MH agar when required. Bacterial pellets and extracted DNA were stored at −20 °C. *C. jejuni* frozen stocks were stored at −80 °C in 50% glycerol.

### Construction of the Tn5 seed library

The Tn5 transposon mutant library of *C. jejuni* NCTC11168 was generated using EZ-Tn5™ < KAN-2 > Tnp Transposome™ Kit (Cat. No. TSM99K2, Epicentre Biotechnologies, Madison, WI, USA) following the manufacturer’s protocol. Briefly, in vitro transposition reactions consisted of 2 μl 10× EZ-Tn5 reaction buffer, 1 μl of transposome complex, 2 μg of chromosomal DNA, and 15 μl of distilled deionized H_2_O that were incubated for 4 h at 37 °C. The transposed DNA was purified after adding 60 μl of distilled deionized H_2_O followed by phenol-chloroform extraction and then ethanol precipitation of DNA. DNA was recovered in 40 μl TE buffer (pH 8.0). Next, in vitro transposed DNA was repaired by adding 40 μl of transposed DNA, 6 μl of T4 DNA polymerase buffer (New England Biolabs, NEB), 4.8 μl of dNTPs mix (2.5 mM), 7.7 μl distilled H_2_O, and 1.5 μl T4 DNA polymerase (1 U/ μl, NEB) and incubating at 11 °C for 20 min in a thermal cycler. The reaction was inactivated by incubating at 75 °C for 15 min. The second repair reaction consisted of a 60 μl reaction mixture (previous reaction), 12 μl T4 DNA ligase buffer (NEB), 1.5 μl T4 DNA ligase (NEB) and 46.5 μl dH_2_O that was incubated overnight at 16 °C. This was followed by DNA dialysis on top of a nitrocellulose membrane floating on 10–20 ml distilled deionized water for 20 min. All of the reaction was used for one transformation of *C. jejuni* NCTC11168 following the natural transformation method described by Davis et al. 2008 (briefly explained in the next section) [25]. Naturally transformed *C. jejuni* NCTC11168 were selected on MH agar plates with TMP and Km. The mutants were scrapped off the plate into 1× PBS, centrifuged, and the pellet was stored at −80 °C. We performed 14 transformations with each producing ~100,000 mutants. An equal number of mutants from each transformation was combined to create Tn5 seed mutant library (the seed library) as shown in Fig. 1.

### Reconstruction of Tn5 libraries

Genomic DNA extracted from the complex Tn5 seed library (Tn5 seed library DNA) was used for reconstruction of Tn5 libraries in the same and different strain backgrounds of *C. jejuni*. The Tn5 seed library was naturally transformed into *C. jejuni* NCTC11168 and *C. jejuni* 81–176 backgrounds following the natural transformation protocol for *C. jejuni* as described by Davis et al. 2008 [51]. Briefly, *C. jejuni* strains from frozen stock were streaked on MH agar containing TMP and incubated for 16 h under microaerophilic conditions at 37 °C. Next day, a heavy inoculum from the plate was streaked on MH agar with TMP and incubated for 16 h. The bacterial growth from the 16 h growth plate was resuspended in 1 ml MH broth without antibiotics and the OD_600_ was adjusted to 0.5 in MH broth. One ml fresh melted MH agar (without antibiotics) was pipetted into a 5 ml plastic test-tube and was allowed to solidify. An aliquot of 0.5 ml bacteria adjusted to OD_600_ was added into a test-tube containing 1 ml of solidified MH agar and mixed gently and incubated for 3 h at 37 °C in microaerophilic conditions (bi-phasic medium). Following this, 500 μg of seed library DNA was added to the biphasic medium and it was incubated for 4 h in the above conditions. The transformants were collected in a microcentrifuge tube, centrifuged for 2 min and resuspended in MH broth. Finally, the transformants were plated directly or after serial dilutions on MH agar plates supplemented with TMP and Km and incubated for 2 days. The colonies were counted from dilution plates and collected from direct plates in 1X PBS, and centrifuged. The supernatant was discarded and the bacterial pellet was stored at −20 °C (Fig. 1).

### Transposon junction amplification and sequencing

Genomic DNA was extracted from the bacterial pellets of complex Tn5 libraries using the QIAamp DNA Mini Kit (Qiagen, Valencia, CA, USA) following the manufacturer’s protocol. Qubit 2.0 Fluorometer (Life Technologies, Carlsbad, CA) was used to check the concentration and purity of extracted DNA. Tn-seq DNA libraries for Illumina sequencing were prepared using the previously developed protocol in our laboratory [52] with minor modifications as described in detail in Additional file 1 (Protocol S1). The first step was a single primer extension step using a primer specific to one end of the transposon. In this study, for each library DNA, the linear extension step was performed either a regular primer, dual priming oligonucleotide (DPO) primer or both. DPO primers were designed as described by Chun et al. (2007) [32] and used to increase the specificity of PCR amplification of the Tn5-chromosome junction sequences. The regular primer (5′-GATCCTCTAGAGTCGACCTGCAGGCATGCA-5′) and DPO primer (5′-ACCGTGGCGGGGATCCTCTAGAGTIIIIITGCAGGCAT-3′) were located 32 bp and 35 bp upstream of the Tn5-genome junction, respectively (Additional file 1: Fig. S4). Briefly, either regular or DPO primers were used for linear extension PCR using GoTaq G2 hot start colorless master mix (Promega Corporation, Madison, USA). The linear extension was followed by addition of a C tail and then exponential PCR to amplify Tn5-genome junction sequences. Then the product was heated at 65 °C for 15 min, mixed with loading buffer and the PCR products were run on 1% agarose gel. DNA from 300 to 500 bp were gel-purified using Zymoclean™ Gel DNA Recovery Kit following the manufacturer’s protocol (Irvine, Ca, USA). The oligonucleotide used in this study are shown in Additional file 1: Table S1. Equal quantities of DNA (10 ng) were mixed for each library and sent for Illumina HiSeq 4000 single end read sequencing with 90 cycles (DNA Technologies Core, UC Davis Genome Center, Davis, CA 95616).

### Data analysis

Illumina sequencing reads were demultiplexed allowing a perfect match of barcode and transposon mosaic end using a custom Perl script (Additional file 1: Script S1). The 22 bp genomic junction sequences were extracted and used for downstream analysis in different manners for (1) the seed library and the library reconstructed in the same *C. jejuni* NCTC11168 strain background, and (2) the library reconstructed in a different background (*C. jejuni* 81–176). For the libraries in the *C. jejuni* NCTC11168 background, the junction sequences were aligned to the complete sequence of the same genome using Bowtie version 2.2.8 [53]. The sequence alignment map (SAM) files were then inputted into EL-ARTIST for the analysis of the essential genome following the user manual [54]. Briefly, the *C. jejuni* NCTC11168 Tn5 library mapped to 400 bp genomic windows and the *C. jejuni* 81–176 Tn5 library to 500 bp genomic windows. Insertion sites were linked to their associated annotated genomic features. There was no obvious insertion bias according to the insertion sites with respect to the replication of origin (Fig. 2a, b, c, and d). Thus, raw data were used for downstream analysis. Then, sliding window analysis was used to define regions with lower read counts, which were used to train a hidden Markov model to predict each window to be essential or non-essential for growth. Because of the differences in read numbers and complexity of the Tn5 library, we used sliding windows of different sizes and different *p*-value thresholds for calling a region significantly underrepresented in reads, appropriate for each Tn5 library (Additional file 2: SH1).

Additionally, we used Tn-seq Explorer to assign the number of unique Tn5 insertions sites and read counts to each gene using Bowtie aligned SAM files [55]. Tn5 insertion read counts and unique insertion sites were only considered in the central 80% of the gene (CDS) excluding insertions from the beginning and end 10% of the gene. While, for transfer RNA (tRNA), ribosomal (rRNA) and pseudogenes, unique insertion and read counts were considered for the whole gene length.

In contrast, to the libraries in the *C. jejuni* 11,168 background, we had to employ a different strategy for the downstream analysis using the 22 bp junction sequences for the library reconstructed in the *C. jejuni* 81–176 background due to the previously known differences in the genomic regions [40]. For this library, genomic DNA from the seed library (*C. jejuni* NCTC 11168) was transferred to *C. jejuni* 81–176 through natural transformation. For the incoming genomic DNA fragments containing Tn5 insertions to integrate into the recipient genome, there must be sufficient homology between the two strains at the DNA sequence level. To determine the homology levels in *C. jejuni* 81–176, DNA sequences flanking 1000 bp upstream and downstream sequences of the coding sequences (CDS) were extracted along with the CDS for all genes in *C. jejuni* NCTC 11168 using a custom Python script (Additional file 1: Script S2) and these NCTC 11168 sequences were BLASTed against *C. jejuni* 81–176 genome using the BLASTn from the command-line interface to display result in tabular output format 6. Single best BLAST hits were kept for each query sequence based on the highest bit score. Then, BLAST output tables were filtered so that genes having the highest homology with flanking sequences were retained. We arbitrarily used the following combination of condition to filter the BLAST tabular output file: alignment over >55% of query length, percent identity ≥98%, mismatches <50 nucleotide and gaps <5 with were kept. Next, the genes of *C. jejuni* NCTC 11168 having the highest probability of homologous recombination in *C. jejuni* 81–176 were searched again for orthologous genes present in *C. jejuni* 81–176. Only the orthologous genes with flanking sequences of *C. jejuni* 81–176 having high homology in *C. jejuni* NCTC 11168 were considered for the analysis of the essential genome (Additional file 3: SH1).

## Additional files

Additional file 1: This pdf file contains supplementary figures (Figures S1-S6), a detailed protocol for DNA library preparation (Protocol S1), Table S1, and Perl and Python scripts (Script S1 and S2). Figure legends are available within file. (PDF 662 kb)

Additional file 2: Essential genes of *C. jejuni* NCTC 11168. This xlsx file contains Microsoft Excel spreadsheet: SH1-SH4. (XLSX 342 kb)

Additional file 3: Essential genes of *C. jejuni* 81–176. This xlsx file contains Microsoft Excel spreadsheet: SH1 and SH2. (XLSX 236 kb)

Additional file 4: Comparative analysis of the essential genome of *C. jejuni* with previously identified essential genes. (XLSX 68 kb)
