# Supplementary material for: Essential genome of Campylobacter jejuni
Source: BMC Genomics. 2017 Aug 14;18:616. doi: 10.1186/s12864-017-4032-8 (PMC5557513; doi:10.1186/s12864-017-4032-8)
Supplement: Supplementary file 1 — This pdf file contains supplementary figures (Figures S1-S6), a detailed protocol for DNA library preparation (Protocol S1), Table S1, and Perl and Python scripts (Script S1 and S2). Figure legends are available within file. (PDF 662 kb) [file 12864_2017_4032_MOESM1_ESM.pdf]

Additional File 1

This pdf file contains supplementary Figures (S1-S6), detailed protocol for DNA library preparation (Protocol S1), and Table S1. Legends for the figures are given below.

Figure S1: Number of unique Tn5 insertion sites in Tn5 mutant libraries of *C. jejuni* NCTC 11168. Number inside the bracket is percentage. Tn5 libraries are named accordingly as in the main body of manuscript.

Figure S2: Essential gene of *C. jejuni* NCTC 11168 identified using EL-Artist pipeline. Figures outside brackets are number of identified essential genes and numbers inside bracket are overall percentage (%). Libraries are named accordingly as in the main body of manuscript.

Figure S3: Assignment of cluster of orthologous group (COG) to essential genes of *C. jejuni* 81-176 (CJ-811176). Essential gene were only analyzed for genes of *C. jejuni* 81-176 that had sufficient homologous sequence in upstream and downstream including coding sequence in the *C. jejuni* NCTC 11168 background (Since Tn5 library in *C. jejuni* 811176 were constructed using genomic DNA of Tn5 library of *C. jejuni* NCTC 11168). Figure at the top of bar indicated the number of essential genes. COG symbol are similar as in the main body of manuscript.

Figure S4: Primer design for linear extension of Tn5 junction sequence during DNA library preparation for Illumina Sequencing. DPO-Tn5-Kn2 primer (green) is dual priming oligonucleotide primer and Ez-Tn5 primer3 (blue) is regular primer. ME: Mosaic end sequence.

Figure S5: Schematic diagram of DNA library preparation of transposon library for Illumina sequencing using DPO primer. Tn-specific primer 1 (DPO-Tn5-Kan2, Supplementary Table1) is used for linear extension and Tn-specific primer 2 (library specific barcoded primer according) is used for exponential PCR in conjunction with C-tail specific primer (HTM-Primer, Supplementary Table 1).

Figure S6: Agarose gel electrophoresis of Tn5 mutant library after exponential PCR. DNA from 300-500 bp is extracted from agarose gel (1%), pooled in equal quantity (10 ng/ sample) and sent for sequencing. [1,2: Tn5 library of *C. jejuni*; 3: Control (genomic DNA of C. jejuni NCTC 11168); and M: Hi-Low DNA Marker).

Figure S1

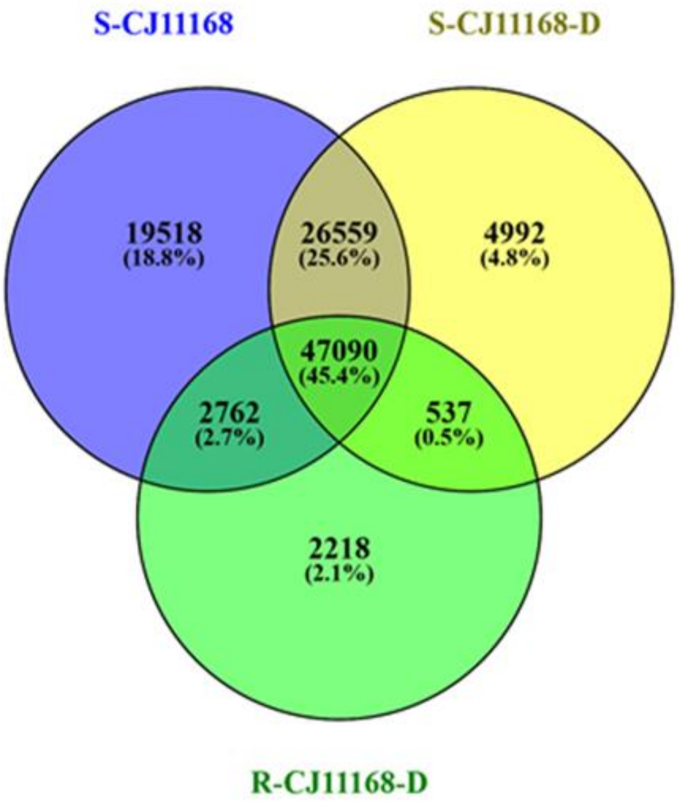
S-CJ11168
S-CJ11168-D
19518
(18.8%)
26559
(25.6%)
4992
(4.8%)
47090
(45.4%)
2762
(2.7%)
537
(0.5%)
2218
(2.1%)
R-CJ11168-D

Figure S2

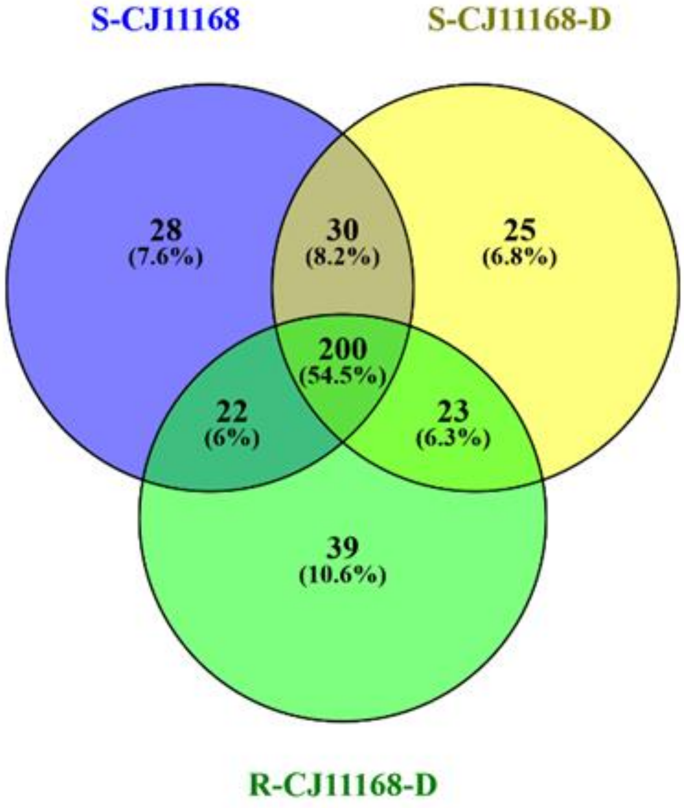
S-CJ11168
S-CJ11168-D
28
(7.6%)
30
(8.2%)
25
(6.8%)
200
(54.5%)
22
(6%)
23
(6.3%)
39
(10.6%)
R-CJ11168-D

Figure S3

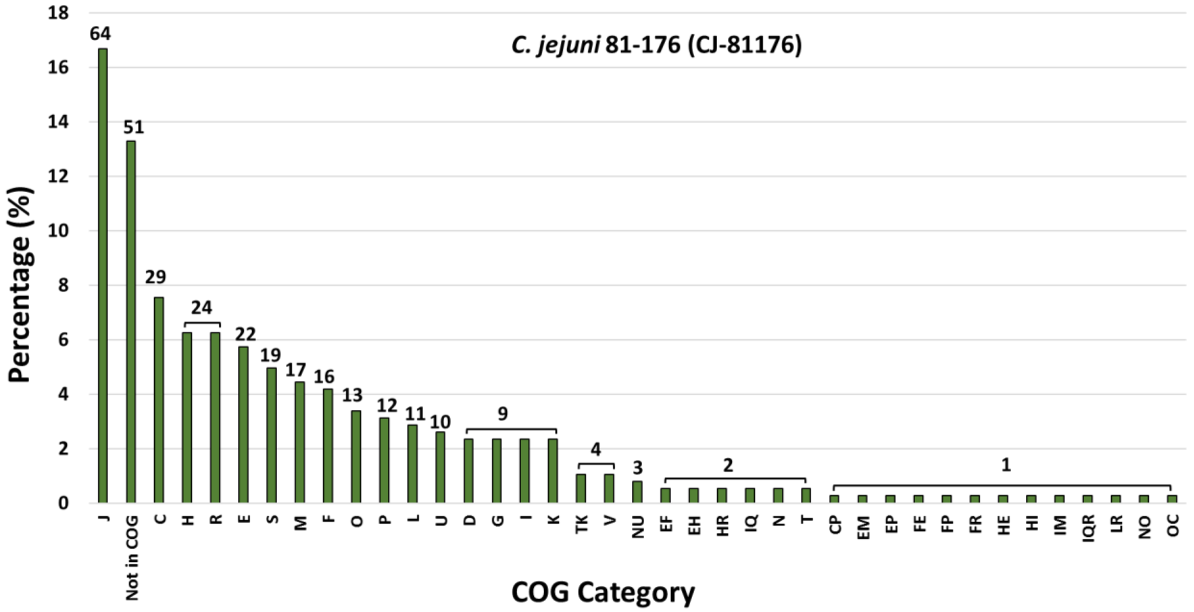
C. jejuni 81-176 (CJ-81176)
Percentage (%)
COG Category
0
2
4
6
8
10
12
14
16
18
64
51
29
24
22
19
17
16
13
12
11
10
9
4
3
2
1
J
Not in COG
C
H
R
E
S
M
F
O
P
L
U
D
G
I
K
TK
V
NU
EF
EH
HR
IQ
N
T
CP
EM
EP
FE
FP
FR
HE
HI
IM
IQR
LR
NO
OC

Figure S4

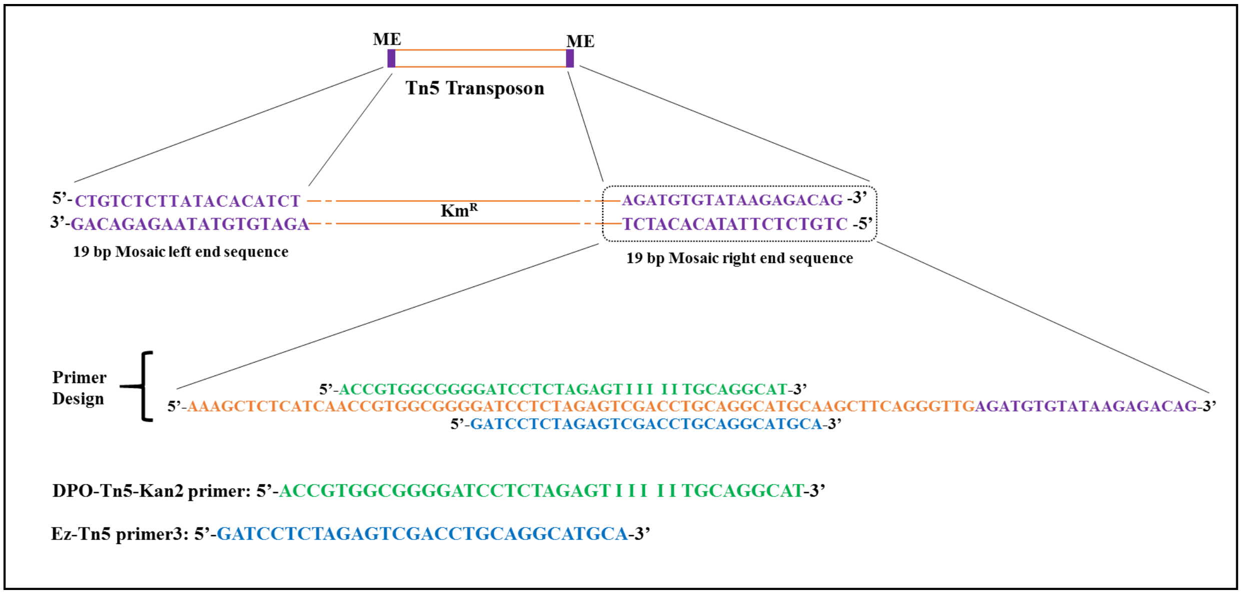
ME
ME
Tn5 Transposon
5'-CTGTCTCTTATACACATCT
3'-GACAGAGAATATGTGTAGA
KmR
AGATGTGTATAAGAGACAG -3'
TCTACACATATTCTCTGTC -5'
19 bp Mosaic left end sequence
19 bp Mosaic right end sequence
Primer Design
5'-ACCGTGGCGGGGATCCTCTAGAGT I I I I I TGCAGGCAT-3'
5'-AAAGCTCTCATCAACCGTGGCGGGGATCCTCTAGAGTCGACCTGCAGGCATGCAAGCTTCAGGGTTGAGATGTGTATAAGAGACAG-3'
5'-GATCCTCTAGAGTCGACCTGCAGGCATGCA-3'
DPO-Tn5-Kan2 primer: 5'-ACCGTGGCGGGGATCCTCTAGAGT I I I I I TGCAGGCAT-3'
Ez-Tn5 primer3: 5'-GATCCTCTAGAGTCGACCTGCAGGCATGCA-3'

Protocol S1:

DNA library preparation protocol for transposon sequencing (Tn-seq) using dual priming oligonucleotide (DPO)

This method of DNA library preparation is improved version of previously developed methodology in our laboratory [1, 2]. A single dual priming oligonucleotide (DPO) primer is used for linear extension of transposon junction [3]. The purified PCR product is subjected to addition of C tail, which is controlled effectively by the mixture of deoxycytidine triphosphate (dCTP) and dideoxy CTP (ddCTP) [4]. Then, the purified C-tailed product is amplified by transposon specific primer and poly G primer as shown in Figure S5.

MATERIALS

DNA extracted from Tn5 mutant library

Wild type DNA (Control DNA)

QIAamp DNA Mini Kit (Qiagen, Valencia, CA, USA)

Qubit 2.0 Fluorometer (Life Technologies, Carlsbad, CA)

Oligonucleotide **(Table S1)**

GoTaq® G2 Hot Start Colorless Master Mix (Promega, WI, USA)

Nuclease-free water

DNA Clean and Concentrator Kit (Zymo Research, Irvine, CA, USA)

Terminal Transferase (TdT, New England Biolabs, Ipswich, MA, USA)

TdT Reaction Buffer (10X)

$CoCl_2$ (2.5 mM) (TdT, New England Biolabs, Ipswich, MA, USA)

dCTP (100 mM) (Promega, Madison, WI, USA)

ddCTP (10 mM) (Promega Madison, WI, USA)

1 % Agarose gel

Zymoclean Gel DNA Recovery Kit (Zymo Research, Irvine, CA, USA)

Thermocycler (PCR machine)

STEPS:

Step 1: Linear extension PCR

1. Reaction mixture

Nuclease free H_2O	22 µl
GoTaq G2 Hot Start Colorless Mx	25 µl
DPO-Tn5-Kan2 (10 µm)	1 µl
Genomic DNA of Tn library (~100ng)	2 µl
Total	50 µl

2. PCR cycle

	95°C	2 min
	[95°C	30 sec]
50 cycles	[63°C	45 sec]
	[72°C	10 sec]
	4°C	hold

Note: Regular primer was used during the linear extension of the seed library.

3. Purify the linear extension PCR products using DNA clean and concentrator kit. Elute DNA in 11µl EB buffer and store at -20°C.

Step 2: C-tailing reaction

1. Preparation of dNTP working stock
   Dilute 100 mM dCTP to 10 mM dCTP with ddH_2O (nuclease-free)
   Dilute 10 mM ddCTPto 1 mM ddCTP with ddH_2O (nuclease-free)

2. Reaction mixture

DNA (linear extension products)	10.0 µl
TdT Buffer (10X)	2.0 µl
2.5 mM $CoCl_2$	2.0 µl
10 mM dCTP	2.4 µl
1 mM ddCTP	1.0 µl
ddH_2O	2.1 µl
Terminal transferase	0.5 µl
Total	20.0 µl

3. Incubate the reaction tube at 37°C for 1 hr.

4. Incubate the reaction tube at 75°C for 20 min for heat inactivation of TdT.

5. Purify the C-tailed products using DNA clean and concentrator kit. Elute DNA in 10 µl EB buffer and store at -20°C.

Step 3: PCR to amplify Tn-flanking sequences

1. Reaction mixture

ddH_2O	22.5 µl
GoTaq G2 Hot Start colorless Mx	25 µl
IR2 BC primer with an unique barcode (10 µM)	1 µl
HTM primer (20 µM)	0.5 µl
C-tailed DNA	1 µl
Total	50 µl

4. PCR cycle

	95°C	2 min
	[95°C	30 sec]
36 cycles	[58°C	45 sec]
	[72°C	20 sec]
	72°C	10 min
	4°C	hold

Step 4: Gel-purification of PCR products

1. Mix the sample with loading buffer and heat at 65ºC for 15 min.
2. Run 10 µl/sample on 1% agarose gel.
3. Cut 300-500bp bands and gel-purify DNA fragments (as shown in Figure S6).

Figure S5

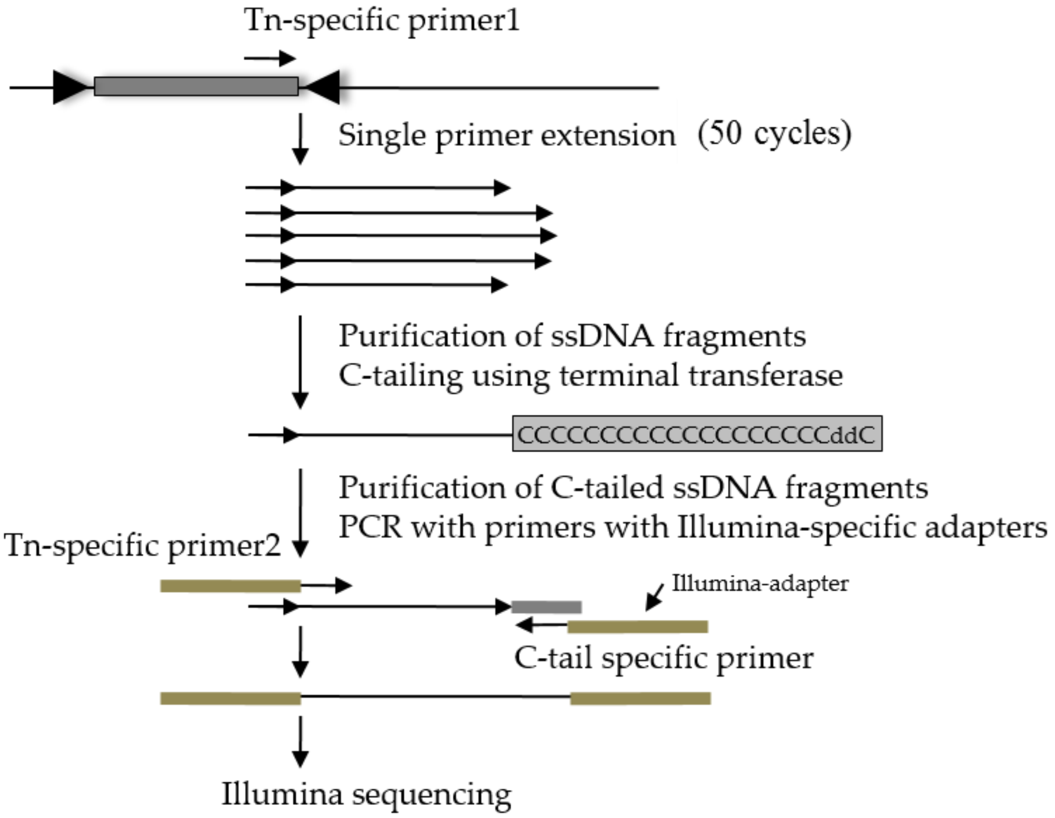

Tn-specific primer1
Single primer extension (50 cycles)
Purification of ssDNA fragments
C-tailing using terminal transferase
CCCCCCCCCCCCCCCCCCCddC
Purification of C-tailed ssDNA fragments
PCR with primers with Illumina-specific adapters
Tn-specific primer2
Illumina-adapter
C-tail specific primer
Illumina sequencing

Figure S6

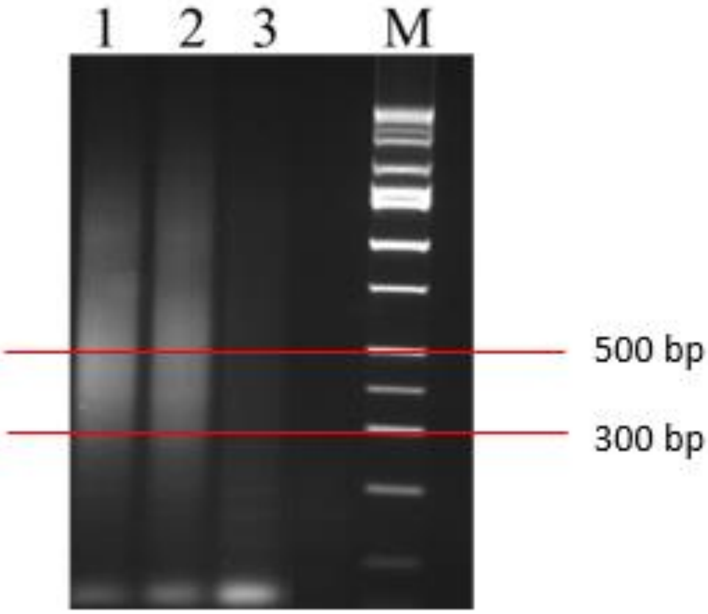

Table S1: List of primers used for DNA library preparation of transposon sequencing.

Primer Name	Sequence (5'-3')	Sample
Ez-Tn5 primer3	5'-GATCCTCTAGAGTCGACCTGCAGGC ATGCA-3'	
DPO-Tn5-Kan2	5'-ACCGTGGCGGGGATCCTCTAGAGTCGA CCTGCAGGCAT-3'	
IR2-IS-B4	5'-AATGATACGGCGACCACCGAGATCTAC ACTCTTTCCCTACACGACGCTCTTCCGA TCTNNNNAGaatgataTCAGGGTTGAGATG TGTATAAGGGACAG-3'	S-CJ11168
IR2-IS-B19	5'-AATGATACGGCGACCACCGAGATCTACA CTCTTTCCCTACACGACGCTCTTCCGATC TNNNNAGatcgacTCAGGGTTGAGATGTGT ATAAGAGACAG-3'	S-CJ11168-D
IR2-IS-B7	5'-AATGATACGGCGACCACCGAGATCTACA CTCTTTCCCTACACGACGCTCTTCCGATC TNNNNAGcagatcTCAGGGTTGAGATGTGT ATAAGAGACAG-3'	R-CJ11168-D
IR2-IS-B8	5'-AATGATACGGCGACCACCGAGATCTACA CTCTTTCCCTACACGACGCTCTTCCGATCT NNNNAGacttgaTCAGGGTTGAGATGTGTAT AAGAGACAG-3'	R-CJ81176-D
HTM-Primer	5'-CAAGCAGAAGACGGCATACGAGCTCTTC CGATCTGGGGGGGGGGGGGGGG-3'	

Ez-Tn5 primer3 (regular primer) and DPO-Tn5-Kan2 (dual priming oligonucleotide) were used for linear extension during DNA library preparation. NNNN: four random nucleotide used for efficient clustering. Nucleotide in small letter are barcode designed to allow sorting of Illumina sequence reads according to sample. Barcoded primer and HTM-primer were used for exponential PCR.

References

1. Dawoud TM, Jiang T, Mandal RK, Ricke SC, Kwon YM: Improving the efficiency of transposon mutagenesis in *Salmonella* Enteritidis by overcoming host-restriction barriers. *Mol Biotechnol* 2014, 56(11)**;**1004-1010.

2. Kwon YM, Ricke SC, Mandal RK: Transposon sequencing: methods and expanding applications. *Appl Microbiol Biotechnol* 2016, 100(1)**;**31-43.

3. Chun JY, Kim KJ, Hwang IT, Kim YJ, Lee DH, Lee IK, Kim JK: Dual priming oligonucleotide system for the multiplex detection of respiratory viruses and SNP genotyping of CYP2C19 gene. *Nucleic Acids Res* 2007, 35(6)**;**e40.

4. Lazinski DW, Camilli A: Homopolymer tail-mediated ligation PCR: a streamlined and highly efficient method for DNA cloning and library construction. *BioTechniques* 2013, 54(1)**;**25-34.
